# Crystal structure of 1-phenyl­imido-1-{6-[1-(phenyl­imino)­eth­yl]pyridin-2-yl}ethan-1-yl-κ^3^ *N*,*N*′,*N*′′)iron(II)

**DOI:** 10.1107/S2056989016015528

**Published:** 2016-10-18

**Authors:** Ka-Cheong Lau, Alexander S. Filatov, Richard F. Jordan

**Affiliations:** a Department of Chemistry, The University of Chicago, 5735 South Ellis ave, Chicago, Il 60637, USA

**Keywords:** crystal structure, bis­(imino)­pyridine ligand, pyridine-di­imine, redox-active, iron, radical anion

## Abstract

The title iron complex, [Fe(C_21_H_19_N_3_)_2_], consists of an Fe^II^ atom chelated by two tridentate bis­(imino)­pyridine radical anions in a slightly distorted octa­hedral coordination environment. In the solid state, there are two independent half-mol­ecules in the asymmetric unit, and the complete mol­ecular structure is formed by applying twofold rotation symmetry with the twofold rotation axis passing through an Fe atom. In the crystal, the Fe-containing complexes are not involved in any particular direct inter­molecular inter­actions, with the shortest C—H_Ar_ contacts between neighboring phenyl groups being *ca* 3.2 Å.

## Abstract 2

In the structure of the iron(II) title compound with tridentate radical anionic 2,6-bis­[1-(phenyl­imino)­eth­yl]pyridine ligands, two independent half-mol­ecules are present in the asymmetric unit.

## Chemical context   

Transition metal complexes that contain bis­(imino)­pyridine ligands are highly active catalysts for olefin oligomerization and polymerization (Small *et al.*, 1998 ▸; Britovsek *et al.*, 1998 ▸, 1999 ▸; Small, 2015 ▸), and many other reactions (for example: Bart *et al.*, 2004 ▸; Tondreau *et al.*, 2012**a* ▸,b* ▸; Obligacion & Chirik, 2013 ▸; Bouwkamp *et al.*, 2006 ▸; Hoyt *et al.*, 2015 ▸; Sylvester & Chirik, 2009 ▸). In pursuit of this chemistry, dicationic iron(II) complexes that are chelated by two neutral bis­(imino)­pyridine ligands have been synthesized and characterized by X-ray diffraction (for example: de Bruin *et al.*, 2000 ▸; Ionkin *et al.*, 2006 ▸). However, until recently, neutral {bis­(imino)­pyrid­ine}_2_Fe complexes were only generated *in situ* and characterized by cyclic voltammetry and electronic spectroscopy (de Bruin *et al.*, 2000 ▸). Thus far, four neutral {bis­(imino)­pyridine}_2_Fe complexes that contain alkyl or functionalized-phenyl substituents on the imine nitro­gen atoms have been crystallographically characterized (Wile *et al.*, 2009 ▸). Here we report the crystal structure of a parent mol­ecule of the class, (PDI)_2_Fe [PDI = 2,6-(C_6_H_5_-N=CMe)_2_-C_5_H_3_N], **1**.
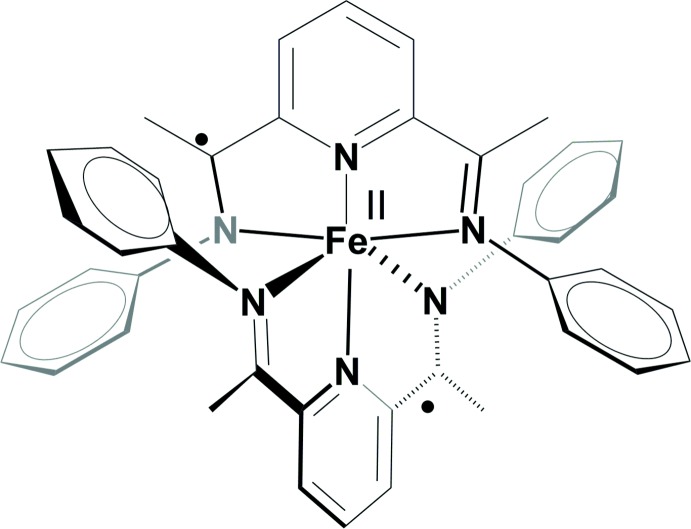


## Structural commentary   

**Figure 1:**
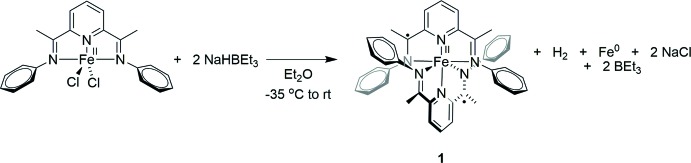
Schematic representation of the synthesis of **1**.

**Figure 2:**
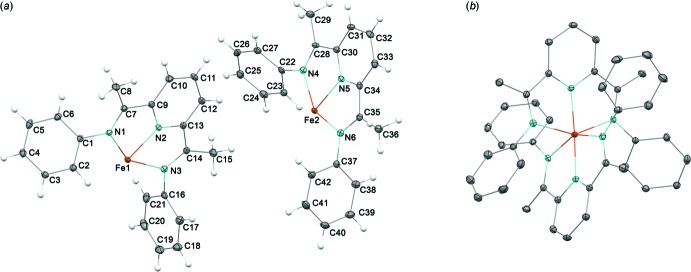
(*a*) The asymmetric unit of **1**, showing the two half-complexes and (*b*) the mol­ecular structure of one of the completed complexes (Fe1) with H atoms omitted for clarity and displacement ellipsoids shown at the 50% probability level.

Complex **1** was synthesized by reduction of (PDI)FeCl_2_ with NaHBEt_3_ (Fig. 1 ▸). Crystals of **1** suitable for X-ray diffraction were obtained from Et_2_O solution. There are two independent mol­ecules in the asymmetric unit (Fig. 2 ▸ *a*). The whole mol­ecular structure is formed by applying twofold rotation symmetry with the twofold rotation axis passing through an Fe atom (Fig. 2 ▸ *b*). The two independent mol­ecules have very similar bond lengths and angles except for the N(imine)—Fe bond lengths (Table 1 ▸). One mol­ecule (Fe2) has two equivalent N(imine)—Fe bond lengths [2.155 (2), 2.157 (2) Å], while the other (Fe1) has two noticeably different N(imine)—Fe bond lengths [2.149 (2), 2.173 (2) Å] (Table 1 ▸). The C—C bond lengths in the pyridine and phenyl rings [1.380 (3)–1.401 (3) Å] and the C—N bond lengths in the pyridine rings [1.366 (3), 1.372 (3) Å] in the two mol­ecules are very similar. The N(imine)—Fe—N(pyridine) angles in the two mol­ecules are also similar [73.85 (8)–75.01 (8)°]. The two chelate planes formed by (PDI)Fe units are almost perpendicular to each other, presumably to avoid steric congestion [92.05 (9) and 93.32 (8) for Fe1- and Fe2-containing complexes, respectively, and measured as a dihedral angle between two planes passing through three nitro­gen atoms of the coordinating PDI ligand].

An analogue of **1** containing a *para*-meth­oxy substituent on the imine-phenyl ring, {2,6-(4-MeO-C_6_H_4_-N=CMe)_2_-C_5_H_3_N}_2_Fe (**2**) was also crystallized with two independent mol­ecules in the asymmetric unit (Wile *et al.*, 2009 ▸), and it is inter­esting to compare the geometric parameters of **1** and **2**. As observed for **1**, the N(imine)—Fe bond lengths in one of the two independent mol­ecules in the asymmetric unit of **2** are similar [2.1278 (19), 2.1481 (19) Å], while those in the other exhibit much greater disparity [2.1159 (19), 2.1711 (19) Å]. Although the electron-donating meth­oxy substituents of **2** are expected to render the imino nitro­gens more basic than those in **1**, the N(imine)—Fe bond lengths in **1** and **2** are very similar [range for **1**: 2.149 (2) – 2.173 (2) Å; range for **2**: 2.1159 (19) – 2.1711 (19) Å].

**Figure 3:**
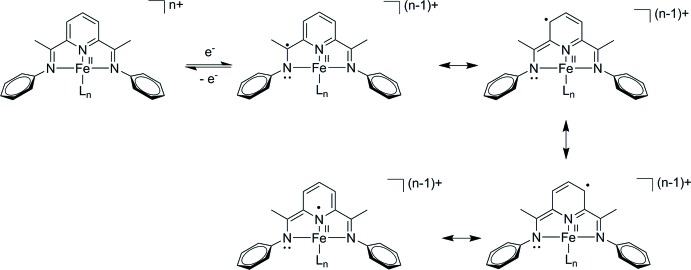
Resonance structures of the mono-reduced ligand in **1**.

Bis(imino)­pyridine ligands are redox-active owing to the extensive π-conjugation (de Bruin *et al.*, 2000 ▸; Budzelaar *et al.*, 2001 ▸; Knijnenburg *et al.*, 2006 ▸). Reduction of the ligand causes characteristic changes in bond lengths, as expected from the resonance structures of the mono-reduced ligand as shown in Fig. 3 ▸ (Bart *et al.*, 2006 ▸). In particular, reduction by 1 e^−^ lengthens the C(imine)—N(imine) bond length from *ca* 1.28 to 1.32 Å and shortens the C(imine)—C(ipso) bond length from *ca* 1.50 to 1.44 Å. In the free ligand, the C(imine)—N(imine) and C(imine)—C(ipso) bond lengths are 1.266 (4) and 1.497 (5) Å (Mentes *et al.*, 2001 ▸). The electronic structure of **2** was shown to consist of an Fe^II^ atom and two mono-reduced bis­(imino)­pyridine radical anions by Mössbauer spectroscopy, magnetic data, crystallographic data and broken-symmetry DFT calculations. The C(imine)—N(imine) [1.294 (3)–1.327 (3) Å] and C(imine)—C(ipso) [1.440 (4)–1.456 (3) Å] bond lengths in **1** are close to those in **2** [C(imine)—N(imine) = 1.306 (3)–1.313 (3) Å and C(imine)—C(ipso) = 1.432 (3)–1.444 (3) Å], consistent with mono-reduced PDI ligands and an Fe^II^ atom as observed for **2**.

## Supra­molecular features   

**Figure 4:**
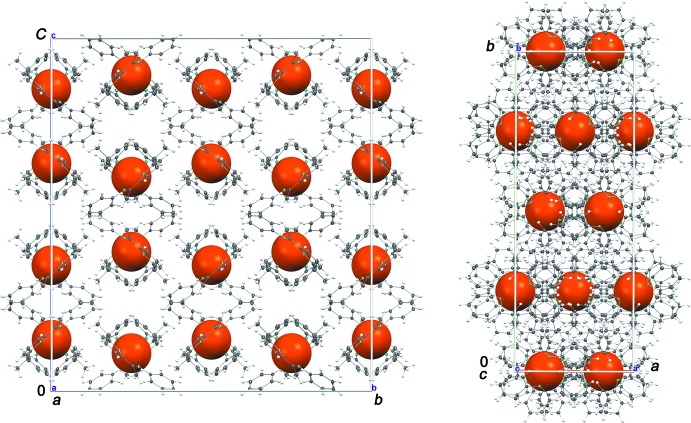
Orthogonal views of the crystal packing of **1** projected along the *a* (left) and *c* (right) axes. Fe atoms are shown as large brown spheres of arbitrary radius.

The structure crystallizes in the ortho­rhom­bic *Ccce* space group (No. 68) with rather large unit-cell parameters (*b* and *c* axes are both greater than 30 Å). Fig. 4 ▸ shows the crystal packing with Fe atoms forming a sub-lattice with ≃ 1/4 of the cell volume. The different relative orientation of ligands around the central Fe atoms leads to the obtained large unit cell. In the crystal, the Fe-containing complexes are not involved in any particular direct inter­molecular inter­actions. The shortest C⋯H_Ar_ contacts with neighboring phenyl groups start at about 3.2 Å.

## Database survey   

A search of the Cambridge Structural Database (CSD, Version 5.37, last update November 2015; Groom *et al.*, 2016 ▸) reveals several crystallographically characterized neutral iron(II) complexes that are chelated by two bis­(imino)­pyridine radical anions [CSD refcodes: DUFCAJ, DUFBOW, DUFCEN, DUFBUC (Wile *et al.*, 2009 ▸)]. Examples containing chromium [CSD refcode: OGUYOG (Wang *et al.*, 2015 ▸)] and molybdenum [CSD refcode: OGUYEW (Wang *et al.*, 2015 ▸)] have also been reported.

## Synthesis and crystallization   

Compound **1** was isolated from the attempted synthesis of (PDI)FeCl by reduction of (PDI)FeCl_2_ with NaHBEt_3_ in Et_2_O. Et_2_O (10 ml) was added to (PDI)FeCl_2_ (0.113 g, 0.26 mmol) in a Schlenk flask to form a purple slurry. A solution of NaHBEt_3_ in Et_2_O (0.065 *M*, 4 ml, 0.26 mmol) was added dropwise at 238 K to the slurry. The mixture was warmed to room temperature (*ca* 293 K) for 1 h and evolved to a red slurry. The mixture was filtered and the filtrate was concentrated under vacuum to afford purple crystals of **1**, which were identified by X-ray crystallographic analysis.

## Refinement   

Crystal data, data collection and structure refinement details are summarized in Table 2 ▸. All carbon-bound H atoms were included in idealized positions for structure factor calculations [C—H = 0.95–0.98 Å, *U*_iso_(H) set to 1.2–1.5*U*_eq_(C)].

## Supplementary Material

Crystal structure: contains datablock(s) I. DOI: 10.1107/S2056989016015528/wm5324sup1.cif

Structure factors: contains datablock(s) I. DOI: 10.1107/S2056989016015528/wm5324Isup2.hkl

Supporting information file. DOI: 10.1107/S2056989016015528/wm5324Isup3.cdx

Supporting information file. DOI: 10.1107/S2056989016015528/wm5324Isup4.cdx

Supporting information file. DOI: 10.1107/S2056989016015528/wm5324Isup5.cdx

CCDC reference: 1507934

Additional supporting information: crystallographic information; 3D view; checkCIF report

## Figures and Tables

**Table 1:** Selected bond lengths (Å)

Fe1—N1	2.149 (2)	N3—C14	1.300 (3)
Fe1—N2	2.028 (2)	C7—C9	1.443 (4)
Fe1—N3	2.173 (2)	C13—C14	1.450 (4)
N1—C7	1.327 (3)	Fe2—N4	2.155 (2)
N2—C9	1.368 (3)	Fe2—N5	2.029 (2)
N2—C13	1.372 (3)	Fe2—N6	2.157 (2)

**Table 2:** Experimental details

Crystal data	
Chemical formula	[Fe(C_21_H_19_N_3_)_2_]
*M*_r_	682.63
Crystal system, space group	Orthorhombic, *C* *c* *c* *e*
Temperature (K)	100
*a*, *b*, *c* (Å)	11.9028 (5), 32.2189 (14), 35.5223 (15)
*V* (Å^3^)	13622.6 (10)
*Z*	16
Radiation type	Mo *K*α
μ (mm^−1^)	0.48
Crystal size (mm)	0.32 × 0.24 × 0.10

Data collection	
Diffractometer	Bruker D8 Venture PHOTON 100 CMOS
Absorption correction	Multi-scan (*SADABS*; Bruker, 2014 ▸)
*T*_min_, *T*_max_	0.662, 0.745
No. of measured, independent and observed [*I* > 2σ(*I*)] reflections	116689, 7006, 5821
*R*_int_	0.062
(sin θ/λ)_max_ (Å^−1^)	0.626

Refinement	
*R*[*F* ^2^ > 2σ(*F* ^2^)], *wR*(*F* ^2^), *S*	0.053, 0.128, 1.19
No. of reflections	7006
No. of parameters	447
H-atom treatment	H-atom parameters constrained
Δρ_max_, Δρ_min_ (e Å^−3^)	0.66, −0.63

Computer programs: *APEX2* and *SAINT* (Bruker, 2014 ▸), *SHELXT2014* (Sheldrick, 2015*a* ▸), *SHELXL2014* (Sheldrick, 2015*b* ▸), *OLEX2* (Dolomanov *et al.*, 2009 ▸), *Mercury* (Macrae *et al.*, 2008 ▸) and *publCIF* (Westrip, 2010 ▸).

## supplementary crystallographic information

### Crystal data

**Table:**

[Fe(C_21_H_19_N_3_)_2_]	*D*_x_ = 1.331 Mg m^−^^3^
*M**_r_* = 682.63	Mo *K*α radiation, λ = 0.71073 Å
Orthorhombic, *C**c**c**e*	Cell parameters from 9834 reflections
*a* = 11.9028 (5) Å	θ = 2.2–26.4°
*b* = 32.2189 (14) Å	µ = 0.48 mm^−^^1^
*c* = 35.5223 (15) Å	*T* = 100 K
*V* = 13622.6 (10) Å^3^	Plate, dark violet
*Z* = 16	0.32 × 0.24 × 0.10 mm
*F*(000) = 5728	

### Data collection

**Table:**

Bruker D8 Venture PHOTON 100 CMOS diffractometer	7006 independent reflections
Radiation source: INCOATEC ImuS micro-focus source	5821 reflections with *I* > 2σ(*I*)
Mirrors monochromator	*R*_int_ = 0.062
Detector resolution: 10.4167 pixels mm^-1^	θ_max_ = 26.4°, θ_min_ = 1.9°
ω and phi scans	*h* = −14→14
Absorption correction: multi-scan (*SADABS*; Bruker, 2014)	*k* = −40→40
*T*_min_ = 0.662, *T*_max_ = 0.745	*l* = −44→44
116689 measured reflections	

### Refinement

**Table:**

Refinement on *F*^2^	Primary atom site location: dual
Least-squares matrix: full	Secondary atom site location: difference Fourier map
*R*[*F*^2^ > 2σ(*F*^2^)] = 0.053	Hydrogen site location: inferred from neighbouring sites
*wR*(*F*^2^) = 0.128	H-atom parameters constrained
*S* = 1.19	*w* = 1/[σ^2^(*F*_o_^2^) + (0.0365*P*)^2^ + 50.0482*P*] where *P* = (*F*_o_^2^ + 2*F*_c_^2^)/3
7006 reflections	(Δ/σ)_max_ = 0.002
447 parameters	Δρ_max_ = 0.66 e Å^−^^3^
0 restraints	Δρ_min_ = −0.63 e Å^−^^3^

### Special details

**Table:**

Geometry. All esds (except the esd in the dihedral angle between two l.s. planes) are estimated using the full covariance matrix. The cell esds are taken into account individually in the estimation of esds in distances, angles and torsion angles; correlations between esds in cell parameters are only used when they are defined by crystal symmetry. An approximate (isotropic) treatment of cell esds is used for estimating esds involving l.s. planes.

### Fractional atomic coordinates and isotropic or equivalent isotropic displacement parameters (Å^2^)

**Table:**

	*x*	*y*	*z*	*U*_iso_*/*U*_eq_	
Fe1	0.5000	0.7500	0.60614 (2)	0.01348 (13)	
N1	0.37427 (17)	0.77288 (6)	0.56801 (6)	0.0146 (4)	
N2	0.36620 (17)	0.71185 (6)	0.61341 (6)	0.0148 (4)	
N3	0.55060 (18)	0.70451 (6)	0.64801 (6)	0.0166 (5)	
C1	0.3933 (2)	0.80895 (8)	0.54604 (7)	0.0151 (5)	
C2	0.4937 (2)	0.81186 (8)	0.52588 (7)	0.0189 (6)	
H2	0.5457	0.7895	0.5264	0.023*	
C3	0.5181 (2)	0.84716 (8)	0.50503 (8)	0.0195 (6)	
H3	0.5861	0.8485	0.4911	0.023*	
C4	0.4444 (2)	0.88047 (8)	0.50431 (8)	0.0205 (6)	
H4	0.4614	0.9045	0.4899	0.025*	
C5	0.3457 (2)	0.87820 (8)	0.52478 (8)	0.0220 (6)	
H5	0.2952	0.9010	0.5247	0.026*	
C6	0.3199 (2)	0.84291 (8)	0.54545 (7)	0.0183 (5)	
H6	0.2517	0.8418	0.5593	0.022*	
C7	0.2742 (2)	0.75478 (8)	0.56928 (7)	0.0169 (5)	
C8	0.1756 (2)	0.76466 (9)	0.54468 (8)	0.0231 (6)	
H8A	0.1223	0.7820	0.5586	0.035*	
H8B	0.1386	0.7388	0.5370	0.035*	
H8C	0.2014	0.7796	0.5223	0.035*	
C9	0.2667 (2)	0.72135 (8)	0.59619 (8)	0.0174 (5)	
C10	0.1672 (2)	0.70106 (8)	0.60645 (8)	0.0230 (6)	
H10	0.0983	0.7080	0.5945	0.028*	
C11	0.1700 (2)	0.67089 (9)	0.63407 (9)	0.0245 (6)	
H11	0.1029	0.6572	0.6414	0.029*	
C12	0.2715 (2)	0.66062 (8)	0.65106 (8)	0.0210 (6)	
H12	0.2747	0.6393	0.6695	0.025*	
C13	0.3675 (2)	0.68179 (8)	0.64075 (7)	0.0168 (5)	
C14	0.4765 (2)	0.67718 (8)	0.65847 (7)	0.0174 (5)	
C15	0.4957 (2)	0.64459 (9)	0.68814 (8)	0.0243 (6)	
H15A	0.5765	0.6418	0.6929	0.036*	
H15B	0.4655	0.6180	0.6794	0.036*	
H15C	0.4577	0.6528	0.7114	0.036*	
C16	0.6627 (2)	0.70349 (8)	0.66146 (7)	0.0182 (5)	
C17	0.7394 (3)	0.67610 (9)	0.64561 (8)	0.0255 (6)	
H17	0.7143	0.6560	0.6279	0.031*	
C18	0.8519 (3)	0.67774 (10)	0.65533 (9)	0.0319 (7)	
H18	0.9033	0.6586	0.6445	0.038*	
C19	0.8899 (3)	0.70702 (10)	0.68070 (10)	0.0335 (7)	
H19	0.9674	0.7084	0.6871	0.040*	
C20	0.8139 (3)	0.73426 (10)	0.69674 (9)	0.0325 (7)	
H20	0.8393	0.7543	0.7144	0.039*	
C21	0.7009 (3)	0.73262 (9)	0.68734 (8)	0.0264 (6)	
H21	0.6495	0.7515	0.6986	0.032*	
Fe2	0.2500	0.5000	0.64429 (2)	0.01289 (13)	
N4	0.12861 (18)	0.52706 (6)	0.68203 (6)	0.0157 (4)	
N5	0.10938 (18)	0.46510 (6)	0.63800 (6)	0.0146 (4)	
N6	0.29045 (18)	0.45369 (6)	0.60258 (6)	0.0160 (4)	
C22	0.1573 (2)	0.56140 (8)	0.70498 (7)	0.0165 (5)	
C23	0.2584 (2)	0.55886 (8)	0.72503 (7)	0.0188 (5)	
H23	0.3041	0.5348	0.7225	0.023*	
C24	0.2929 (2)	0.59069 (8)	0.74837 (8)	0.0225 (6)	
H24	0.3605	0.5879	0.7623	0.027*	
C25	0.2294 (2)	0.62673 (9)	0.75159 (8)	0.0236 (6)	
H25	0.2529	0.6486	0.7677	0.028*	
C26	0.1311 (2)	0.63013 (8)	0.73093 (8)	0.0229 (6)	
H26	0.0877	0.6548	0.7326	0.027*	
C27	0.0946 (2)	0.59791 (8)	0.70769 (8)	0.0201 (6)	
H27	0.0271	0.6008	0.6937	0.024*	
C28	0.0248 (2)	0.51224 (8)	0.68000 (7)	0.0163 (5)	
C29	−0.0753 (2)	0.52709 (9)	0.70198 (8)	0.0252 (6)	
H29A	−0.0498	0.5432	0.7238	0.038*	
H29B	−0.1190	0.5031	0.7106	0.038*	
H29C	−0.1224	0.5446	0.6859	0.038*	
C30	0.0117 (2)	0.47772 (8)	0.65463 (7)	0.0170 (5)	
C31	−0.0902 (2)	0.45885 (8)	0.64529 (8)	0.0211 (6)	
H31	−0.1579	0.4679	0.6568	0.025*	
C32	−0.0925 (2)	0.42691 (9)	0.61925 (8)	0.0241 (6)	
H32	−0.1617	0.4139	0.6129	0.029*	
C33	0.0070 (2)	0.41386 (8)	0.60236 (8)	0.0212 (6)	
H33	0.0071	0.3916	0.5848	0.025*	
C34	0.1056 (2)	0.43407 (8)	0.61172 (7)	0.0159 (5)	
C35	0.2133 (2)	0.42730 (8)	0.59329 (7)	0.0161 (5)	
C36	0.2259 (2)	0.39396 (9)	0.56407 (8)	0.0223 (6)	
H36A	0.1913	0.4031	0.5405	0.033*	
H36B	0.1889	0.3686	0.5728	0.033*	
H36C	0.3059	0.3884	0.5599	0.033*	
C37	0.4006 (2)	0.45267 (8)	0.58710 (7)	0.0178 (5)	
C38	0.4804 (2)	0.42575 (9)	0.60131 (9)	0.0264 (6)	
H38	0.4595	0.4054	0.6194	0.032*	
C39	0.5910 (3)	0.42826 (10)	0.58929 (10)	0.0360 (8)	
H39	0.6451	0.4094	0.5990	0.043*	
C40	0.6235 (3)	0.45784 (10)	0.56331 (10)	0.0362 (8)	
H40	0.6997	0.4599	0.5556	0.043*	
C41	0.5432 (3)	0.48453 (10)	0.54870 (9)	0.0324 (7)	
H41	0.5642	0.5047	0.5305	0.039*	
C42	0.4326 (2)	0.48196 (9)	0.56043 (8)	0.0233 (6)	
H42	0.3782	0.5003	0.5502	0.028*	

### Atomic displacement parameters (Å^2^)

**Table:**

	*U*^11^	*U*^22^	*U*^33^	*U*^12^	*U*^13^	*U*^23^
Fe1	0.0106 (2)	0.0120 (2)	0.0179 (3)	−0.00086 (19)	0.000	0.000
N1	0.0102 (10)	0.0139 (10)	0.0197 (11)	0.0024 (8)	−0.0003 (8)	−0.0004 (9)
N2	0.0115 (10)	0.0120 (10)	0.0210 (11)	−0.0015 (8)	0.0005 (8)	−0.0009 (8)
N3	0.0149 (11)	0.0154 (11)	0.0194 (11)	0.0006 (9)	−0.0002 (9)	0.0012 (9)
C1	0.0151 (13)	0.0135 (12)	0.0166 (12)	−0.0002 (10)	−0.0038 (10)	−0.0016 (10)
C2	0.0190 (13)	0.0157 (12)	0.0218 (14)	0.0002 (11)	−0.0017 (11)	−0.0009 (11)
C3	0.0148 (13)	0.0206 (13)	0.0231 (14)	−0.0023 (11)	0.0020 (11)	−0.0007 (11)
C4	0.0219 (14)	0.0163 (13)	0.0234 (14)	−0.0011 (11)	−0.0051 (11)	0.0033 (11)
C5	0.0268 (15)	0.0160 (13)	0.0231 (14)	0.0051 (11)	−0.0076 (12)	−0.0016 (11)
C6	0.0173 (13)	0.0180 (13)	0.0195 (13)	0.0021 (11)	−0.0031 (10)	−0.0023 (10)
C7	0.0133 (12)	0.0144 (12)	0.0229 (13)	0.0005 (10)	−0.0015 (10)	−0.0027 (10)
C8	0.0154 (13)	0.0231 (14)	0.0307 (15)	−0.0027 (11)	−0.0060 (11)	0.0029 (12)
C9	0.0140 (13)	0.0151 (12)	0.0232 (14)	0.0007 (10)	0.0005 (10)	−0.0027 (10)
C10	0.0150 (13)	0.0198 (13)	0.0342 (16)	0.0016 (11)	−0.0037 (12)	0.0000 (12)
C11	0.0156 (14)	0.0202 (14)	0.0376 (17)	−0.0058 (11)	0.0060 (12)	0.0019 (12)
C12	0.0176 (14)	0.0169 (13)	0.0285 (15)	−0.0014 (10)	0.0049 (11)	0.0031 (11)
C13	0.0158 (13)	0.0134 (12)	0.0211 (13)	0.0004 (10)	0.0017 (10)	0.0001 (10)
C14	0.0185 (14)	0.0136 (12)	0.0200 (13)	0.0010 (10)	0.0019 (10)	0.0007 (10)
C15	0.0204 (14)	0.0265 (15)	0.0259 (14)	−0.0016 (12)	−0.0016 (12)	0.0095 (12)
C16	0.0172 (13)	0.0171 (13)	0.0202 (13)	−0.0036 (10)	−0.0024 (10)	0.0070 (10)
C17	0.0252 (15)	0.0243 (14)	0.0271 (15)	0.0012 (12)	0.0004 (12)	0.0013 (12)
C18	0.0180 (15)	0.0352 (17)	0.0423 (18)	0.0016 (13)	−0.0011 (13)	0.0077 (15)
C19	0.0189 (15)	0.0325 (17)	0.049 (2)	−0.0028 (13)	−0.0091 (14)	0.0144 (15)
C20	0.0326 (17)	0.0264 (15)	0.0385 (18)	−0.0112 (13)	−0.0160 (14)	0.0052 (13)
C21	0.0310 (16)	0.0194 (14)	0.0286 (15)	0.0013 (12)	−0.0071 (13)	0.0032 (12)
Fe2	0.0107 (2)	0.0120 (2)	0.0160 (3)	−0.0018 (2)	0.000	0.000
N4	0.0151 (11)	0.0149 (10)	0.0171 (11)	0.0001 (8)	−0.0001 (9)	0.0002 (9)
N5	0.0135 (11)	0.0132 (10)	0.0171 (11)	−0.0028 (8)	−0.0001 (8)	0.0004 (8)
N6	0.0163 (11)	0.0145 (10)	0.0173 (11)	0.0005 (9)	0.0004 (9)	0.0000 (9)
C22	0.0166 (13)	0.0171 (12)	0.0157 (12)	−0.0052 (10)	0.0056 (10)	−0.0002 (10)
C23	0.0177 (13)	0.0174 (13)	0.0212 (14)	−0.0019 (11)	0.0031 (11)	−0.0019 (11)
C24	0.0204 (14)	0.0246 (14)	0.0226 (14)	−0.0050 (12)	0.0012 (11)	−0.0014 (12)
C25	0.0259 (15)	0.0192 (13)	0.0257 (14)	−0.0047 (12)	0.0055 (12)	−0.0066 (12)
C26	0.0255 (15)	0.0154 (13)	0.0277 (15)	0.0020 (11)	0.0117 (12)	−0.0027 (11)
C27	0.0184 (13)	0.0206 (14)	0.0211 (13)	−0.0019 (11)	0.0048 (11)	0.0012 (11)
C28	0.0087 (12)	0.0189 (13)	0.0212 (13)	−0.0001 (10)	0.0018 (10)	0.0008 (11)
C29	0.0168 (14)	0.0267 (15)	0.0320 (16)	−0.0031 (11)	0.0057 (12)	−0.0091 (12)
C30	0.0133 (12)	0.0171 (12)	0.0206 (13)	−0.0008 (10)	0.0011 (10)	0.0021 (11)
C31	0.0112 (13)	0.0215 (14)	0.0305 (15)	0.0008 (11)	0.0021 (11)	−0.0007 (12)
C32	0.0169 (14)	0.0226 (14)	0.0327 (16)	−0.0058 (11)	−0.0046 (12)	−0.0011 (12)
C33	0.0230 (14)	0.0177 (13)	0.0228 (14)	−0.0026 (11)	−0.0015 (11)	−0.0026 (11)
C34	0.0182 (13)	0.0135 (12)	0.0159 (12)	−0.0004 (10)	−0.0016 (10)	0.0019 (10)
C35	0.0172 (13)	0.0150 (12)	0.0163 (12)	0.0001 (10)	−0.0015 (10)	−0.0002 (10)
C36	0.0194 (14)	0.0229 (14)	0.0246 (14)	−0.0020 (11)	0.0011 (11)	−0.0071 (12)
C37	0.0174 (13)	0.0155 (12)	0.0204 (13)	−0.0019 (10)	0.0012 (11)	−0.0067 (10)
C38	0.0186 (15)	0.0249 (15)	0.0358 (17)	−0.0002 (11)	0.0018 (12)	0.0015 (13)
C39	0.0168 (15)	0.0318 (17)	0.059 (2)	0.0034 (13)	0.0021 (15)	−0.0043 (16)
C40	0.0194 (15)	0.0300 (17)	0.059 (2)	−0.0070 (13)	0.0152 (15)	−0.0159 (16)
C41	0.0331 (17)	0.0243 (15)	0.0399 (18)	−0.0087 (13)	0.0187 (14)	−0.0054 (14)
C42	0.0226 (15)	0.0195 (13)	0.0280 (15)	0.0003 (11)	0.0067 (12)	−0.0030 (12)

### Geometric parameters (Å, º)

**Table:**

Fe1—N1^i^	2.149 (2)	Fe2—N4	2.155 (2)
Fe1—N1	2.149 (2)	Fe2—N4^ii^	2.155 (2)
Fe1—N2	2.028 (2)	Fe2—N5^ii^	2.029 (2)
Fe1—N2^i^	2.028 (2)	Fe2—N5	2.029 (2)
Fe1—N3^i^	2.173 (2)	Fe2—N6^ii^	2.157 (2)
Fe1—N3	2.173 (2)	Fe2—N6	2.157 (2)
N1—C1	1.418 (3)	N4—C22	1.416 (3)
N1—C7	1.327 (3)	N4—C28	1.327 (3)
N2—C9	1.368 (3)	N5—C30	1.366 (3)
N2—C13	1.372 (3)	N5—C34	1.368 (3)
N3—C14	1.300 (3)	N6—C35	1.294 (3)
N3—C16	1.418 (3)	N6—C37	1.422 (3)
C1—C2	1.396 (4)	C22—C23	1.400 (4)
C1—C6	1.401 (4)	C22—C27	1.397 (4)
C2—H2	0.9500	C23—H23	0.9500
C2—C3	1.388 (4)	C23—C24	1.381 (4)
C3—H3	0.9500	C24—H24	0.9500
C3—C4	1.386 (4)	C24—C25	1.390 (4)
C4—H4	0.9500	C25—H25	0.9500
C4—C5	1.383 (4)	C25—C26	1.385 (4)
C5—H5	0.9500	C26—H26	0.9500
C5—C6	1.388 (4)	C26—C27	1.395 (4)
C6—H6	0.9500	C27—H27	0.9500
C7—C8	1.497 (4)	C28—C29	1.502 (4)
C7—C9	1.443 (4)	C28—C30	1.440 (4)
C8—H8A	0.9800	C29—H29A	0.9800
C8—H8B	0.9800	C29—H29B	0.9800
C8—H8C	0.9800	C29—H29C	0.9800
C9—C10	1.401 (4)	C30—C31	1.397 (4)
C10—H10	0.9500	C31—H31	0.9500
C10—C11	1.382 (4)	C31—C32	1.384 (4)
C11—H11	0.9500	C32—H32	0.9500
C11—C12	1.390 (4)	C32—C33	1.393 (4)
C12—H12	0.9500	C33—H33	0.9500
C12—C13	1.381 (4)	C33—C34	1.383 (4)
C13—C14	1.450 (4)	C34—C35	1.456 (4)
C14—C15	1.505 (4)	C35—C36	1.501 (4)
C15—H15A	0.9800	C36—H36A	0.9800
C15—H15B	0.9800	C36—H36B	0.9800
C15—H15C	0.9800	C36—H36C	0.9800
C16—C17	1.389 (4)	C37—C38	1.382 (4)
C16—C21	1.390 (4)	C37—C42	1.391 (4)
C17—H17	0.9500	C38—H38	0.9500
C17—C18	1.384 (4)	C38—C39	1.386 (4)
C18—H18	0.9500	C39—H39	0.9500
C18—C19	1.381 (5)	C39—C40	1.382 (5)
C19—H19	0.9500	C40—H40	0.9500
C19—C20	1.384 (5)	C40—C41	1.387 (5)
C20—H20	0.9500	C41—H41	0.9500
C20—C21	1.386 (4)	C41—C42	1.383 (4)
C21—H21	0.9500	C42—H42	0.9500
			
N1—Fe1—N1^i^	101.86 (11)	N4—Fe2—N4^ii^	103.07 (11)
N1^i^—Fe1—N3	90.40 (8)	N4—Fe2—N6^ii^	89.87 (8)
N1—Fe1—N3	148.84 (8)	N4—Fe2—N6	148.95 (8)
N1—Fe1—N3^i^	90.40 (8)	N4^ii^—Fe2—N6^ii^	148.95 (8)
N1^i^—Fe1—N3^i^	148.84 (8)	N4^ii^—Fe2—N6	89.87 (8)
N2—Fe1—N1^i^	114.79 (8)	N5^ii^—Fe2—N4^ii^	74.91 (8)
N2^i^—Fe1—N1	114.79 (8)	N5^ii^—Fe2—N4	113.41 (8)
N2—Fe1—N1	75.01 (8)	N5—Fe2—N4^ii^	113.41 (8)
N2^i^—Fe1—N1^i^	75.01 (8)	N5—Fe2—N4	74.91 (8)
N2^i^—Fe1—N2	165.36 (12)	N5^ii^—Fe2—N5	167.36 (12)
N2—Fe1—N3^i^	95.96 (8)	N5—Fe2—N6^ii^	97.10 (8)
N2—Fe1—N3	73.84 (8)	N5—Fe2—N6	74.04 (8)
N2^i^—Fe1—N3	95.96 (8)	N5^ii^—Fe2—N6^ii^	74.05 (8)
N2^i^—Fe1—N3^i^	73.84 (8)	N5^ii^—Fe2—N6	97.10 (8)
N3^i^—Fe1—N3	93.62 (11)	N6—Fe2—N6^ii^	93.24 (11)
C1—N1—Fe1	121.09 (16)	C22—N4—Fe2	120.83 (16)
C7—N1—Fe1	116.93 (17)	C28—N4—Fe2	116.42 (17)
C7—N1—C1	121.5 (2)	C28—N4—C22	122.5 (2)
C9—N2—Fe1	119.18 (17)	C30—N5—Fe2	119.30 (17)
C9—N2—C13	119.0 (2)	C30—N5—C34	119.0 (2)
C13—N2—Fe1	120.60 (17)	C34—N5—Fe2	120.49 (17)
C14—N3—Fe1	117.66 (18)	C35—N6—Fe2	118.10 (18)
C14—N3—C16	121.8 (2)	C35—N6—C37	122.7 (2)
C16—N3—Fe1	120.47 (16)	C37—N6—Fe2	119.16 (16)
C2—C1—N1	118.3 (2)	C23—C22—N4	117.0 (2)
C2—C1—C6	118.3 (2)	C27—C22—N4	124.7 (2)
C6—C1—N1	123.3 (2)	C27—C22—C23	118.2 (2)
C1—C2—H2	119.7	C22—C23—H23	119.4
C3—C2—C1	120.5 (2)	C24—C23—C22	121.1 (3)
C3—C2—H2	119.7	C24—C23—H23	119.4
C2—C3—H3	119.6	C23—C24—H24	119.7
C4—C3—C2	120.8 (3)	C23—C24—C25	120.5 (3)
C4—C3—H3	119.6	C25—C24—H24	119.7
C3—C4—H4	120.4	C24—C25—H25	120.6
C5—C4—C3	119.1 (3)	C26—C25—C24	118.8 (3)
C5—C4—H4	120.4	C26—C25—H25	120.6
C4—C5—H5	119.7	C25—C26—H26	119.4
C4—C5—C6	120.7 (3)	C25—C26—C27	121.1 (3)
C6—C5—H5	119.7	C27—C26—H26	119.4
C1—C6—H6	119.7	C22—C27—H27	120.0
C5—C6—C1	120.6 (3)	C26—C27—C22	120.1 (3)
C5—C6—H6	119.7	C26—C27—H27	120.0
N1—C7—C8	126.2 (2)	N4—C28—C29	126.6 (2)
N1—C7—C9	114.0 (2)	N4—C28—C30	114.4 (2)
C9—C7—C8	119.8 (2)	C30—C28—C29	119.0 (2)
C7—C8—H8A	109.5	C28—C29—H29A	109.5
C7—C8—H8B	109.5	C28—C29—H29B	109.5
C7—C8—H8C	109.5	C28—C29—H29C	109.5
H8A—C8—H8B	109.5	H29A—C29—H29B	109.5
H8A—C8—H8C	109.5	H29A—C29—H29C	109.5
H8B—C8—H8C	109.5	H29B—C29—H29C	109.5
N2—C9—C7	114.2 (2)	N5—C30—C28	114.1 (2)
N2—C9—C10	120.8 (2)	N5—C30—C31	120.4 (2)
C10—C9—C7	125.0 (2)	C31—C30—C28	125.4 (2)
C9—C10—H10	120.3	C30—C31—H31	120.0
C11—C10—C9	119.5 (3)	C32—C31—C30	120.0 (3)
C11—C10—H10	120.3	C32—C31—H31	120.0
C10—C11—H11	120.1	C31—C32—H32	120.1
C10—C11—C12	119.8 (3)	C31—C32—C33	119.7 (3)
C12—C11—H11	120.1	C33—C32—H32	120.1
C11—C12—H12	120.4	C32—C33—H33	120.8
C13—C12—C11	119.1 (2)	C34—C33—C32	118.4 (2)
C13—C12—H12	120.4	C34—C33—H33	120.8
N2—C13—C12	121.8 (2)	N5—C34—C33	122.4 (2)
N2—C13—C14	113.0 (2)	N5—C34—C35	112.8 (2)
C12—C13—C14	125.1 (2)	C33—C34—C35	124.6 (2)
N3—C14—C13	114.4 (2)	N6—C35—C34	114.3 (2)
N3—C14—C15	124.7 (2)	N6—C35—C36	125.1 (2)
C13—C14—C15	120.7 (2)	C34—C35—C36	120.4 (2)
C14—C15—H15A	109.5	C35—C36—H36A	109.5
C14—C15—H15B	109.5	C35—C36—H36B	109.5
C14—C15—H15C	109.5	C35—C36—H36C	109.5
H15A—C15—H15B	109.5	H36A—C36—H36B	109.5
H15A—C15—H15C	109.5	H36A—C36—H36C	109.5
H15B—C15—H15C	109.5	H36B—C36—H36C	109.5
C17—C16—N3	119.8 (2)	C38—C37—N6	120.5 (2)
C17—C16—C21	118.8 (3)	C38—C37—C42	119.1 (3)
C21—C16—N3	121.0 (3)	C42—C37—N6	120.1 (2)
C16—C17—H17	119.7	C37—C38—H38	119.9
C18—C17—C16	120.7 (3)	C37—C38—C39	120.3 (3)
C18—C17—H17	119.7	C39—C38—H38	119.9
C17—C18—H18	119.8	C38—C39—H39	119.6
C19—C18—C17	120.4 (3)	C40—C39—C38	120.8 (3)
C19—C18—H18	119.8	C40—C39—H39	119.6
C18—C19—H19	120.4	C39—C40—H40	120.5
C18—C19—C20	119.2 (3)	C39—C40—C41	119.0 (3)
C20—C19—H19	120.4	C41—C40—H40	120.5
C19—C20—H20	119.6	C40—C41—H41	119.8
C19—C20—C21	120.8 (3)	C42—C41—C40	120.4 (3)
C21—C20—H20	119.6	C42—C41—H41	119.8
C16—C21—H21	119.9	C37—C42—H42	119.8
C20—C21—C16	120.1 (3)	C41—C42—C37	120.5 (3)
C20—C21—H21	119.9	C41—C42—H42	119.8
			
Fe1—N1—C1—C2	49.0 (3)	Fe2—N4—C22—C23	−47.6 (3)
Fe1—N1—C1—C6	−126.8 (2)	Fe2—N4—C22—C27	129.6 (2)
Fe1—N1—C7—C8	−178.0 (2)	Fe2—N4—C28—C29	−177.2 (2)
Fe1—N1—C7—C9	−1.2 (3)	Fe2—N4—C28—C30	4.7 (3)
Fe1—N2—C9—C7	9.7 (3)	Fe2—N5—C30—C28	−8.9 (3)
Fe1—N2—C9—C10	−166.8 (2)	Fe2—N5—C30—C31	168.2 (2)
Fe1—N2—C13—C12	167.8 (2)	Fe2—N5—C34—C33	−169.7 (2)
Fe1—N2—C13—C14	−8.5 (3)	Fe2—N5—C34—C35	6.0 (3)
Fe1—N3—C14—C13	−1.3 (3)	Fe2—N6—C35—C34	2.6 (3)
Fe1—N3—C14—C15	−177.5 (2)	Fe2—N6—C35—C36	178.1 (2)
Fe1—N3—C16—C17	−96.2 (3)	Fe2—N6—C37—C38	96.8 (3)
Fe1—N3—C16—C21	76.4 (3)	Fe2—N6—C37—C42	−75.9 (3)
N1—C1—C2—C3	−177.7 (2)	N4—C22—C23—C24	−179.5 (2)
N1—C1—C6—C5	176.9 (2)	N4—C22—C27—C26	−179.3 (2)
N1—C7—C9—N2	−5.2 (3)	N4—C28—C30—N5	2.3 (3)
N1—C7—C9—C10	171.1 (3)	N4—C28—C30—C31	−174.6 (3)
N2—C9—C10—C11	−0.5 (4)	N5—C30—C31—C32	0.5 (4)
N2—C13—C14—N3	5.9 (3)	N5—C34—C35—N6	−5.4 (3)
N2—C13—C14—C15	−177.7 (2)	N5—C34—C35—C36	178.9 (2)
N3—C16—C17—C18	172.8 (3)	N6—C37—C38—C39	−172.3 (3)
N3—C16—C21—C20	−172.3 (3)	N6—C37—C42—C41	171.9 (3)
C1—N1—C7—C8	9.8 (4)	C22—N4—C28—C29	−3.0 (4)
C1—N1—C7—C9	−173.4 (2)	C22—N4—C28—C30	178.9 (2)
C1—C2—C3—C4	1.1 (4)	C22—C23—C24—C25	−2.1 (4)
C2—C1—C6—C5	1.1 (4)	C23—C22—C27—C26	−2.2 (4)
C2—C3—C4—C5	0.1 (4)	C23—C24—C25—C26	−0.1 (4)
C3—C4—C5—C6	−0.8 (4)	C24—C25—C26—C27	1.0 (4)
C4—C5—C6—C1	0.2 (4)	C25—C26—C27—C22	0.1 (4)
C6—C1—C2—C3	−1.7 (4)	C27—C22—C23—C24	3.2 (4)
C7—N1—C1—C2	−139.1 (3)	C28—N4—C22—C23	138.5 (3)
C7—N1—C1—C6	45.1 (4)	C28—N4—C22—C27	−44.3 (4)
C7—C9—C10—C11	−176.6 (3)	C28—C30—C31—C32	177.3 (3)
C8—C7—C9—N2	171.9 (2)	C29—C28—C30—N5	−175.9 (2)
C8—C7—C9—C10	−11.8 (4)	C29—C28—C30—C31	7.2 (4)
C9—N2—C13—C12	0.7 (4)	C30—N5—C34—C33	−2.5 (4)
C9—N2—C13—C14	−175.6 (2)	C30—N5—C34—C35	173.2 (2)
C9—C10—C11—C12	−0.8 (4)	C30—C31—C32—C33	−0.3 (4)
C10—C11—C12—C13	2.0 (4)	C31—C32—C33—C34	−1.3 (4)
C11—C12—C13—N2	−1.9 (4)	C32—C33—C34—N5	2.8 (4)
C11—C12—C13—C14	173.9 (3)	C32—C33—C34—C35	−172.5 (3)
C12—C13—C14—N3	−170.2 (3)	C33—C34—C35—N6	170.2 (2)
C12—C13—C14—C15	6.2 (4)	C33—C34—C35—C36	−5.5 (4)
C13—N2—C9—C7	177.1 (2)	C34—N5—C30—C28	−176.3 (2)
C13—N2—C9—C10	0.6 (4)	C34—N5—C30—C31	0.8 (4)
C14—N3—C16—C17	79.8 (3)	C35—N6—C37—C38	−81.6 (3)
C14—N3—C16—C21	−107.5 (3)	C35—N6—C37—C42	105.6 (3)
C16—N3—C14—C13	−177.5 (2)	C37—N6—C35—C34	−178.9 (2)
C16—N3—C14—C15	6.3 (4)	C37—N6—C35—C36	−3.4 (4)
C16—C17—C18—C19	−0.7 (5)	C37—C38—C39—C40	0.7 (5)
C17—C16—C21—C20	0.5 (4)	C38—C37—C42—C41	−1.0 (4)
C17—C18—C19—C20	1.0 (5)	C38—C39—C40—C41	−1.4 (5)
C18—C19—C20—C21	−0.6 (5)	C39—C40—C41—C42	1.0 (5)
C19—C20—C21—C16	−0.2 (5)	C40—C41—C42—C37	0.2 (5)
C21—C16—C17—C18	−0.1 (4)	C42—C37—C38—C39	0.5 (4)

Symmetry codes: (i) −*x*+1, −*y*+3/2, *z*; (ii) −*x*+1/2, −*y*+1, *z*.

## References

[bb1] Bart, S. C., Chłopek, K., Bill, E., Bouwkamp, M. W., Lobkovsky, E., Neese, F., Wieghardt, K. & Chirik, P. J. (2006). *J. Am. Chem. Soc.* **128**, 13901–13912. 10.1021/ja064557b 17044718

[bb2] Bart, S. C., Lobkovsky, E. & Chirik, P. J. (2004). *J. Am. Chem. Soc.* **126**, 13794–13807. 10.1021/ja046753t 15493939

[bb3] Bouwkamp, M. W., Bowman, A. C., Lobkovsky, E. & Chirik, P. J. (2006). *J. Am. Chem. Soc.* **128**, 13340–13341. 10.1021/ja064711u 17031930

[bb4] Britovsek, G. J. P., Bruce, M., Gibson, V. C., Kimberley, B. S., Maddox, P. J., Mastroianni, S., McTavish, S. J., Redshaw, C., Solan, G. A., Strömberg, S., White, A. J. P. & Williams, D. J. (1999). *J. Am. Chem. Soc.* **121**, 8728–8740.

[bb5] Britovsek, G. J. P., Gibson, V. C., McTavish, S. J., Solan, G. A., White, A. J. P., Williams, D. J., Britovsek, G. J. P., Kimberley, B. S. & Maddox, P. J. (1998). *Chem. Commun.* pp. 849–850.

[bb6] Bruin, B. de, Bill, E., Bothe, E., Weyhermüller, T. & Wieghardt, K. (2000). *Inorg. Chem.* **39**, 2936–2947. 10.1021/ic000113j 11232835

[bb7] Bruker (2014). *APEX2*, *SAINT*, and *SADABS*. Bruker AXS Inc., Madison, Wisconsin, USA.

[bb8] Budzelaar, P. H. M., de Bruin, B., Gal, A. W., Wieghardt, K. & van Lenthe, J. H. (2001). *Inorg. Chem.* **40**, 4649–4655. 10.1021/ic001457c 11511211

[bb9] Dolomanov, O. V., Bourhis, L. J., Gildea, R. J., Howard, J. A. K. & Puschmann, H. (2009). *J. Appl. Cryst.* **42**, 339–341.

[bb10] Groom, C. R., Bruno, I. J., Lightfoot, M. P. & Ward, S. C. (2016). *Acta Cryst.* B**72**, 171–179. 10.1107/S2052520616003954 PMC4822653 27048719

[bb11] Hoyt, J. M., Schmidt, V. A., Tondreau, A. M. & Chirik, P. J. (2015). *Science*, **349**, 960–963. 10.1126/science.aac7440 26315433

[bb12] Ionkin, A. S., Marshall, W. J., Adelman, D. J., Fones, B. B., Fish, B. M. & Schiffhauer, M. F. (2006). *Organometallics*, **25**, 2978–2992.

[bb13] Knijnenburg, Q., Gambarotta, S. & Budzelaar, P. H. M. (2006). *Dalton Trans.* pp. 5442–5448. 10.1039/b612251e 17117213

[bb14] Macrae, C. F., Bruno, I. J., Chisholm, J. A., Edgington, P. R., McCabe, P., Pidcock, E., Rodriguez-Monge, L., Taylor, R., van de Streek, J. & Wood, P. A. (2008). *J. Appl. Cryst.* **41**, 466–470.

[bb15] Mentes, A., Fawcett, J. & Kemmitt, R. D. W. (2001). *Acta Cryst.* E**57**, o424–o425.

[bb16] Obligacion, J. V. & Chirik, P. (2013). *Org. Lett.* **15**, 2680–2683. 10.1021/ol400990u 23688021

[bb17] Sheldrick, G. M. (2015*a*). *Acta Cryst.* A**71**, 3–8.

[bb18] Sheldrick, G. M. (2015*b*). *Acta Cryst.* C**71**, 3–8.

[bb19] Small, B. L. (2015). *Acc. Chem. Res.* **48**, 2599–2611. 10.1021/acs.accounts.5b00252 26267011

[bb20] Small, B. L., Brookhart, M. & Bennett, A. M. A. (1998). *J. Am. Chem. Soc.* **120**, 4049–4050.

[bb21] Sylvester, K. T. & Chirik, P. J. (2009). *J. Am. Chem. Soc.* **131**, 8772–8774. 10.1021/ja902478p 19552448

[bb22] Tondreau, A. M., Atienza, C. C. H., Darmon, J. M., Milsmann, C., Hoyt, H. M., Weller, K. J., Nye, S. A., Lewis, K. M., Boyer, J., Delis, J. G. P., Lobkovsky, E. & Chirik, P. J. (2012*b*). *Organometallics*, **31**, 4886–4893.

[bb23] Tondreau, A. M., Atienza, C. C. H., Weller, K. J., Nye, S. A., Lewis, K. M., Delis, J. G. P. & Chirik, P. J. (2012*a*). *Science*, **335**, 567–570. 10.1126/science.1214451 22301315

[bb24] Wang, M., Weyhermüller, T. & Wieghardt, K. (2015). *Eur. J. Inorg. Chem.* pp. 3246–3254.

[bb25] Westrip, S. P. (2010). *J. Appl. Cryst.* **43**, 920–925.

[bb26] Wile, B. M., Trovitch, R. J., Bart, S. C., Tondreau, A. M., Lobkovsky, E., Milsmann, C., Bill, E., Wieghardt, K. & Chirik, P. J. (2009). *Inorg. Chem.* **48**, 4190–4200. 10.1021/ic801623m 19035761

